# Hemp Seeds (*Cannabis sativa* L.) in Cattle Nutrition: Effects on Beef Fatty Acid, Vitamin, and Mineral Profiles

**DOI:** 10.3390/ani16193006

**Published:** 2026-09-24

**Authors:** Paulina Pogorzelska-Przybyłek, Zenon Nogalski, Cezary Purwin

**Affiliations:** Department of Animal Nutrition, Feed Science and Cattle Breeding, Faculty of Animal Bioengineering, University of Warmia and Mazury in Olsztyn, Oczapowskiego 5, 10-719 Olsztyn, Poland; zena@uwm.edu.pl (Z.N.); purwin@uwm.edu.pl (C.P.)

**Keywords:** α-linolenic acid, α-tocopherol, dietary supplementation, Holstein–Friesian bulls, intramuscular fat, long-chain n-3 polyunsaturated fatty acids, muscle-specific responses, nutritional quality, retinol

## Abstract

The study aimed to determine the effects of dietary supplementation with whole hemp seeds (*Cannabis sativa* L.) on the nutritional value and health-promoting properties of beef, including the fatty acid profile and the content of vitamins and minerals in muscles. Twenty-four young Holstein–Friesian (HF) bulls were allocated to three feeding groups: a control group (CG) and experimental groups receiving concentrate supplemented with 10% (HS–10) or 17% (HS–17) whole hemp seeds. The concentrations of fatty acids, retinol, α-tocopherol, and selected macronutrients and micronutrients were determined in the longissimus lumborum and semimembranosus muscles. Dietary supplementation with whole hemp seeds increased the concentrations of zinc, magnesium, iron, retinol, and α-tocopherol in muscle tissue, and modified the fatty acid profile by decreasing the proportion of saturated fatty acids (SFAs) and increasing the proportion of unsaturated fatty acids (UFAs) and the PUFA/SFA ratio. However, it did not increase the concentrations of long-chain n-3 PUFAs. The values of most parameters improved in response to 10% hemp seeds, whereas increasing their inclusion level to 17% did not result in any further improvement. Whole hemp seeds can be a valuable component of beef cattle diets, enhancing selected quality attributes and the nutritional value of beef.

## Simple Summary

Consumers are increasingly interested in foods with improved nutritional value, while there is growing interest in using hemp seeds as a feed ingredient for livestock. This study examined whether adding whole hemp seeds to the diets of young bulls could improve the vitamin, mineral, and fat composition of beef. Twenty-four bulls received feed containing no hemp seeds, 10% hemp seeds, or 17% hemp seeds for 91 days. Meat from two muscles was then analyzed. Feeding hemp seeds increased the amounts of zinc, magnesium, iron, vitamin A, and vitamin E in the meat. It also reduced the proportion of saturated fats and increased the proportion of unsaturated fats, resulting in a more favorable fat composition. However, hemp seeds did not increase the amounts of beneficial long-chain omega-3 fats in beef. Most improvements were already achieved with the 10% addition, and raising the level to 17% provided no further clear benefit. These findings show that whole hemp seeds can be a useful cattle-feed ingredient for improving selected nutritional properties of beef, although their ability to enrich beef with long-chain omega-3 fats is limited.

## 1. Introduction

*Cannabis sativa* L. is an annual herbaceous plant cultivated worldwide, and this collective name is used to describe various hemp strains [1]. Historically, hemp was grown primarily to produce fiber from its stems [2]. Hemp seeds were used for therapeutic purposes and in the pharmaceutical and chemical industries [3], whereas cannabinoid-containing flowers were used for medicinal, spiritual/religious, and recreational purposes [4]. In addition to the traditional use of fiber in the textile and paper industries, hemp is now increasingly cultivated for the production of functional foods, animal feed, and medicinal products [1,5]. Industrial hemp cultivated in the European Union (EU) countries has to be listed in the Common Catalogue of Varieties of Agricultural Plant Species of the European Union (EU). Currently, 116 hemp varieties are registered, all of which must contain less than 0.3% delta-9-tetrahydrocannabinol (THC) on a dry matter basis [6]. In recent years, the area under hemp cultivation in the EU has increased substantially from 20,540 hectares (ha) in 2015 to 33,020 ha in 2020, marking an increase of 60%. In the same period, hemp production rose by 84.3%, from 97,130 to 179,020 tons [7]. This expansion is driven by hemp’s beneficial impact on the environment and its use in the production of animal feed and food with a high nutritional value [8].

Meat is a rich source of minerals, including zinc (Zn), iron (Fe), copper (Cu), and selenium (Se). Plant-derived foods often contain only small amounts of these minerals or provide them in forms that are less bioavailable to humans [9]. Dietary mineral deficiencies and the associated health disorders remain a widespread global problem, including in developed countries [10,11]. Beef is a valuable dietary source of minerals, particularly Zn and Fe [12]. Meat contains mostly heme iron bound to myoglobin and hemoglobin, which nearly doubles its bioavailability compared with iron from plant-based foods. In muscle tissues, the highest iron concentrations have been reported in veal (1.63–3.26 mg/100 g) and beef (1.34–2.53 mg/100 g) [13]. Ruminants are often exposed to high Fe intake through drinking water, soil ingestion, or Fe-rich feed. Zinc is also an essential mineral that is required for human health and plays important roles in cell division, tissue repair, and growth [14]. Beef is the richest source of dietary Zn (4.19 mg/100 g). Zinc concentrations are lower in pork (2.6 mg/100 g) and chicken meat (0.50 mg/100 g) [11,13,15,16,17]. Zinc is also present in plant-based foods, but its concentrations are generally lower than those found in foods of animal origin. The Zn and Cu content of bovine tissues has attracted research interest due to the low bioavailability of these trace elements in ruminant nutrition and the widespread prevalence of Zn and Cu deficiencies worldwide [18,19]. Hemp seeds are rich in macronutrients, including potassium (K, 4.63–28.2 g/kg), phosphorus (P, 11.2–11.6 g/kg), calcium (Ca, 1.44–9.55 g/kg), magnesium (Mg, 2.37–6.94 g/kg), and sodium (Na, 0.05–0.45 g/kg). They also contain substantial amounts of trace minerals (micronutrients), including manganese (Mn, 63–110 mg/kg), Zn (42.0–94.0 mg/kg), Cu (10.0–12.0 mg/kg), and Fe (1.13–2.40 mg/kg) [1]. Hemp seed oil is abundant in tocopherols, including γ-tocopherol (89.11%), α-tocopherol (5.66%), β-tocopherol (0.33%), and δ-tocopherol (4.90%) [20], as well as provitamin A, which can be readily converted to vitamin A by animals and humans [21,22].

We hypothesized that the inclusion of whole hemp seeds in the diets of young bulls would modify the fatty acid profile of intramuscular fat and increase the concentrations of selected vitamins and minerals in beef, with the magnitude of these responses potentially differing between inclusion levels and muscle types. The significance of this study lies in its simultaneous evaluation of lipid composition, retinol, α-tocopherol, and selected mineral concentrations in two anatomically distinct muscles, providing a more comprehensive assessment of the nutritional effects of whole hemp seed supplementation.

The aim of this study was to determine the potential effects of dietary supplementation with whole hemp seeds (*Cannabis sativa* L.) on selected indicators of the nutritional value and the health-promoting properties of beef from young Holstein–Friesian (HF) bulls, including the content of vitamins, selected macronutrients and micronutrients, and the fatty acid profile of intramuscular fat (IMF).

## 2. Materials and Methods

### 2.1. Animals and Feeding

The research did not require the approval of a local ethics committee. Nevertheless, throughout the study, the welfare of the animals was ensured in compliance with the Act of 15 January 2015 on the Protection of Animals Used for Scientific or Educational Purposes [23]. The experiment was performed on 24 young Holstein–Friesian (HF) bulls reared at the Agricultural Experiment Station in Bałcyny (53°35′29″ N; 19°50′58″ E; Polska). Calves were housed in a conventional system and were fed a milk replacer and hay supplemented with concentrate. Semi-intensive fattening was started at five months of age. The calves were kept in group pens and were fed *ad libitum* a total mixed ration (TMR) composed of maize silage and grass silage supplemented with concentrate at 2 kg. At the age of approximately 719 days and an average live weight (LW) of 621.7 kg, the animals were divided into three feeding groups of eight animals each using the analogue method. They were placed in three separate pens (20 × 13 m) with deep bedding in a monitored feeding facility, where they had enough space to move around freely. During a two-week adaptation period, the calves were fed *ad libitum* maize silage, which was supplemented with increasing concentrate levels. The feeding trial lasted 91 days. Over this period, the animals received maize silage *ad libitum*, dosed from a self-propelled feed cart, and concentrate at 5 kg per day. The feed ration was offered twice daily in feed bunks, at 9:00 a.m. and 3:00 p.m. The concentrate was composed of ground triticale grain, rapeseed meal, a mineral–vitamin premix, and hemp seeds (*Cannabis sativa* L.). The dietary inclusion levels of hemp seeds varied across dietary treatments: CG—control group fed a diet without hemp seed supplementation; HS10—experimental group fed concentrate supplemented with 10% hemp seeds; HS17—experimental group fed concentrate supplemented with 17% hemp seeds (Table 1 and Table 2). Consequently, each dietary treatment was represented by one pen. Each pen was equipped with a Roughage Intake Control system (RIC, Insentec, Marknesse, The Netherlands), comprising three maize silage feeding stations and two concentrate feeding stations. The system enabled individual identification of animals and recording of fed *ad libitum* maize silage and concentrate intake for each bull. Thus, feed intake was measured at the individual animal level, although the dietary treatments were assigned at the pen level. Mean daily DMI was calculated for each bull from its individual intake records, and treatment means were subsequently calculated from the individual-animal means. Mean DMI of maize silage, concentrate, and the total diet is presented in Table 2. The animals had free access to water and salt licks. The animals remained under veterinary care throughout the experiment, and none of them exhibited any signs of health issues.

### 2.2. Feed Sampling and Analyses

Feed samples were collected once a week to determine the content of basic nutrients [24] as well as neutral detergent fiber (NDF) and acid detergent fiber (ADF) using the method proposed by Van Soest et al. [25]. The pH of silage samples was measured with an HI 8314 pH-meter (Hanna Instruments Poland, Olsztyn, Poland). Silage samples were also analyzed for the concentrations of lactic acid, acetic acid, and butyric acid in aqueous extracts by high-performance liquid chromatography (HPLC; Shimadzu, Kyoto, Japan) with a Varian MetaCarb 67H P/N 5244 column (Varian, Palo Alto, CA, USA). The N-NH3 content of silage samples was determined by direct distillation on the Kjeltec 2100 Distillation Unit (Foss Analytical A/S, Hillerod, Denmark). The energy value of the feeds (UFV) and protein values (PDIN and PDIE) were calculated using INRAtion Prevalim ver.3.3 software (INRA, Paris, France) [26].

#### 2.2.1. Determination of β-Carotene and α-Tocopherol

The content of β-carotene and tocopherols was determined in fresh feed samples (immediately after collection) under limited exposure to sunlight. The samples were extracted with a mixture of petroleum ether and ethanol (96%) (1:1 *v*/*v*) at room temperature, in the dark, for 18 h. The resultant extracts were saponified with a 10% ethanolic KOH solution for two hours in the dark, under a nitrogen atmosphere, using a vortex (IKA Poland Sp. z o. o., Warsaw, Poland), and extracted with ethyl ether and then repeatedly with petroleum ether. The obtained extracts were rinsed with a 10% aqueous NaCl solution and repeatedly with deionized water. The eluates were dehydrated with anhydrous sodium sulfate and then evaporated to dryness at 40 °C on a rotary evaporator (Janke & Kunkel IKA-Labortechnik, Staufen, Germany). The residue was dissolved in 5 cm^3^ of hexane. The α-tocopherol content of feed samples was determined by reversed-phase high-performance liquid chromatography (RP-HPLC; Shimadzu, Kyoto, Japan). A Nucleosil C18 column was used; the mobile phase was methanol: H_2_O (95: 5 *v*/*v*) at a flow rate of 1 cm^3^/min. An RF Ex 293 and Em 326 detector and the (±)-α-tocopherol (DL-all-rac α-tocopherol) external standard (SIGMA-ALDRICH, St. Louis, MI, USA) [27] were used. To determine β-carotene concentration, hexane-extracted feed samples were separated by HPLC with a Gemini 5 μm column (Phenomenex, CA, USA), C18, 110 Å, 250 × 4 mm; the mobile phase was acetonitrile:methanol:dichloromethane (750:200:50 *v*/*v*/*v*; HPLC SIGMA-ALDRICH, St. Louis, MI, USA) at a flow rate of 1 cm^3^/min, UV–vis 450 nm detector, 20 μL loop, external standard—β-carotene type I, synthetic (SIGMA-ALDRICH, St. Louis, MI, USA) [28].

#### 2.2.2. Determination of the Fatty Acid Profile

Fat was extracted by the Soxhlet method [24]. Fatty acids were separated and determined by gas chromatography (GC) in a gas chromatograph (CP-3800, Varian, Walnut Creek, CA, USA). Fatty acid methyl esters (FAMEs) were prepared according to the modified Peisker method (methanol:chloroform:concentrated sulfuric acid, 100:100:1, *v*/*v*) [29]. The resulting FAMEs were stored in sealed tubes and were analyzed by gas chromatography-flame ionization detection (GC-FID; column: 50 m × 0.25 mm × 0.25 μm). The temperature of the GC injection port was set to 225 °C in split mode (split ratio 50:1) with helium as the carrier gas at a constant flow rate of 1.2 mL/min. The detector temperature was 250 °C, and the column temperature was 200 °C. Fatty acids were identified by comparing their retention times with those of pure FAME standards (SIGMA-ALDRICH, St. Louis, MI, USA) and peaks in the analyzed samples. The relative content of fatty acids was expressed as the percentage of the total surface area of all fatty acids detected in each sample.

### 2.3. Meat Sampling and Analysis

The bulls were slaughtered in accordance with Council Regulation (EC) No 1099/2009 of 24 September 2009 on the Protection of Animals at the Time of Slaughter [30] and Annex IV “Methods of Killing Animals” to Directive 2010/63/EU of the European Parliament and of the Council of 22 September 2010 on the Protection of Animals Used for Scientific Purposes [31]. To determine the content of minerals and vitamins in meat, samples were collected from right half-carcasses: samples of the *longissimus lumborum* (LL) muscle were collected between the first and the third lumbar vertebrae (*n* = 24) and samples of the *semimembranosus muscle* (SM) were collected from the center of the muscle (*n* = 24) 96 h post mortem. Meat samples (approx. 300 g each) were packaged individually in polyamide/polyethylene vacuum bags at an ambient temperature of approximately 4 °C, under standard industrial conditions. The samples were transported in a portable cooler box to the research laboratory of the Department of Animal Nutrition, Feed Science, and Cattle Breeding at the University of Warmia and Mazury in Olsztyn; delivery time was approximately 1 h. At the laboratory, the samples were frozen and stored at a temperature of −20 °C until analysis (approx. four months). Prior to analysis, the samples were thawed at a temperature of 4 ± 1 °C for 48 h.

#### 2.3.1. Determination of Fat Content and the Fatty Acid Profile

Fat was extracted from ground meat (Ultra-Turrax, Janke & Kunkel) samples using the Soxhlet method, with hexane as the solvent, in the Buechi B-811 extraction system. The crude fat content of meat and the percentages of fatty acids were determined in accordance with the following standards: PN-EN ISO 5509 [32] and PN-EN ISO 5508 [33]. Fatty acid methyl esters were obtained by dissolving the extracted fat in a methanol–chloroform–H_2_SO_4_ mixture, followed by methylation according to the modified Peisker method [29]. The percentages of 31 fatty acids were determined by GC, using a Varian CP 3800 system fitted with a split/splitless injector and a flame-ionization detector. Samples (1 mL) of FAMEs were transferred to a CP-Sil 88 capillary column (length: 100 m, inner diameter: 0.25 mm). The results were processed using the Galaxie Chromatography Data System. The fatty acids were identified by comparing their retention times with those of commercially available reference standards purchased from Supelco Inc. (Bellefonte, PA, USA). Samples and reference standards were analyzed under identical conditions, i.e., carrier gas—helium, injector temperature—260 °C, detector temperature—260 °C and initial oven temperature—110 °C, raised to 249 °C. The fatty acids were categorized as follows: saturated fatty acids (SFAs), unsaturated fatty acids (UFAs), including monounsaturated fatty acids (MUFAs) and polyunsaturated fatty acids (PUFAs). The following ratios were calculated: UFA/SFA, PUFA/SFA, and n-6/n-3 PUFA. The fatty acid content was expressed as a relative percentage of total fatty acids.

#### 2.3.2. Determination of Mineral Content

Samples of both muscles were homogenized, approximately 0.5 g of the material was weighed into Teflon vessels, and 7 mL of 65% nitric acid (Merck, Darmstadt, Germany) was added. Each sample was tested in duplicate. The vessels were sealed, and the samples were mineralized in a MARS Xpress 5 microwave oven (Candela, Marlborough, MA, USA). Each mineralization session involved two blank samples and two samples of certified reference material. All samples were mineralized and analyzed in duplicate. The mineralization program was as follows: (1) 800 W, 100% power, ramping up to 140 °C over 10 min, then holding this temperature for five minutes; (2) 800 W, 100% power, ramping up to 200 °C, then holding this temperature for 15 min. The mineralized material was cooled and quantitatively transferred to 25 mL volumetric flasks. Minerals were analyzed using an atomic absorption spectrometer (Candela, USA) equipped with light sources for each element to be determined (K, Na, Mg, Zn, and Fe). The elements analyzed by atomic absorption spectroscopy and the operating conditions of the spectrometer are presented in Table 3.

#### 2.3.3. Determination of Vitamin A and Vitamin E Content

The content of vitamins A and E was determined based on the applicable standards [34,35], which were somewhat modified for the needs of this study. Samples of LL and SM muscles were ground, and 2 g specimens were placed in 25 mL amber glass flasks. The samples were saponified at a temperature of 80 °C for 30 min in 1.2 mL of 20% ascorbic acid, 0.6 mL of methanol and 5 mL of 60% potassium hydroxide. After cooling, fat was extracted twice with n-hexane, and the extract was evaporated to dryness in a stream of nitrogen. The fat remaining in the flasks was dissolved in 1 mL of anhydrous ethanol and filtered into amber glass vials. Chemical compounds were separated by HPLC using a silica column. The separated compounds were identified by two detectors in tandem (UV-visible photodiode array detector and fluorescence detector). Alpha-tocopherol was detected by fluorescence spectroscopy, and retinol was detected using the UV–visible photodiode array detector. The injection volume was 20 µL. The content of total α-tocopherol and retinol in meat was calculated in duplicate for each muscle, based on the external standards, from a standard curve of peak area vs. concentration.

### 2.4. Statistical Analysis

The data were analyzed statistically using Statistica software Version 13.3 [36]. The effects of experimental feeding (CG, HS–10 and HS–17) and muscle type (LL and SM) on intramuscular fat content, fatty acid composition, and vitamin and mineral concentrations in beef were determined by the least squares method using the following model:

*Y_ijk_* = *μ* + *A_i_* + *B_j_* + (*AB*)*_ij_* + *e_ijk_* (1)

where *Y_ijk_* is the value of the analyzed parameter, *μ* is the population mean, *A_i_* is the effect of experimental feeding (CG, HS–10 and HS–17), *B_j_* is the effect of muscle type (LL and SM), (*AB*)*_ij_* is the experimental feeding × muscle type interaction (EF × MT), and *e_ijk_* is random error. The differences between means were estimated by Fisher’s test. The significance level was set at *p* ≤ 0.05 and *p* ≤ 0.01.

Meat composition was recorded at the individual-animal level. However, each dietary treatment was assigned to a single pen, and independent pen-level replication was not available. Consequently, the statistical comparisons between dietary treatments were based on individual-animal observations and did not account for potential pen-level effects.

## 3. Results and Discussion

Mean daily maize silage DMI was 7.224, 8.771, and 8.629 kg DM/day in CG, HS–10, and HS–17, respectively, whereas concentrate DMI was similar across all groups (4.255–4.256 kg DM/day; Table 2). The comparable maize silage DMI and virtually identical concentrate DMI in HS–10 and HS–17 indicate that the lack of further improvement in the measured traits at 17% hemp seed inclusion was not associated with reduced feed intake, particularly maize silage intake.

### 3.1. Mineral Content of Bull Muscles

The inclusion of whole hemp seeds in beef cattle diets significantly affected the content of Zn, Mg, and Fe in the analyzed muscles, whereas it had no influence on the mean concentrations of K and Na (Table 4). The Zn content of muscle tissue increased by approximately 24.2% and 29.8% in groups HS–10 and HS–17, respectively, relative to CG. The observed effect suggests that the addition of whole hemp seeds to concentrate could promote Zn retention in muscle tissue. This observation is consistent with the results of experiments in which dietary supplementation with highly available micronutrient sources increased Zn concentration in bovine muscles [37]. In the present study, the Mg content of muscle tissue increased from 208.63 mg/kg in CG to 240.24 mg/kg in group HS–10 and to 246.55 mg/kg in group HS–17 (*p* = 0.001). The values noted in both experimental groups were similar, indicating that the bulls’ response to the applied dietary treatments was not dose-dependent. A previous study involving small ruminants (goats) revealed that increasing dietary inclusion levels of Mg induced a linear increase in its blood and liver concentrations, with no significant effect on its concentration in the longissimus thoracis et lumborum muscle [38]. The animals’ response to an increased supply of Mg may vary across tissues, and it does not reflect a simple relationship between the level of this mineral in feed and its concentration in muscle tissue.

The concentrations of Zn, K, and Na varied significantly between the examined muscles. Higher micronutrient levels in certain muscles may be associated with the specific metabolic activity of these muscles, their vascularization, fiber composition, and the abundance of metal-binding proteins, as well as the proportions of muscle, connective, and adipose tissues in the body. According to the literature, the mineral content of beef may also be modified by cattle breed, muscle type, and the age, growth rate, and diet of the animals, and the differences between tissues do not necessarily reflect mineral supply alone [10,13].

A significant EF × MT interaction was observed for the K content of muscle tissue (Figure 1), despite the absence of the EF effect. This relationship indicates that the effect of the applied dietary treatments on K concentration varied depending on the type of tissue analyzed. This observation does not constitute direct evidence that hemp seeds increased K retention in the LL muscle or reduced it in the SM muscle, since K is the key intracellular cation in skeletal muscles and its concentration in the tissue is strictly regulated by homeostasis. Furthermore, it is influenced not only by nutrition, but also by water content and distribution, the ratio of intracellular space to extracellular space, and muscle fiber composition [39,40]. The mechanism responsible for the observed interactions remains unknown and requires further research.

### 3.2. Retinol and α-Tocopherol Content of Bull Muscles

Hemp seeds fed to young bulls significantly increased the concentrations of retinol and α-tocopherol in their muscles (Table 5). The mean retinol concentration was 0.0059 mg/100 g in CG, 0.013 mg/100 g in group HS–10, and 0.018 mg/100 g in group HS–17 (*p* = 0.001), marking an increase of approximately 120–130% and 190–205%, respectively, in experimental groups relative to CG. The observed effect was not related to muscle type (*p* = 0.190), and the EF × MT interaction was not significant (*p* = 0.555). This indicates that the observed change in retinol content was systemic and was not affected by the metabolic properties of the LL and SM muscles. The increase in retinol concentration may be attributed to the fact that the supply of compounds exhibiting provitamin A activity increased with increasing dietary inclusion levels of hemp seeds. The β-carotene content of feed was 5.69 mg/kg fresh matter (FM) and exceeded that of ground triticale grain and rapeseed meal, which were partially replaced with hemp seeds in diets HS–10 and HS–17. It should be noted, however, that the total supply of β-carotene could also be affected by the intake of maize silage, which contained more β-carotene than hemp seeds (18.20 mg/kg FM). The observed increase in the retinol content of meat could be the outcome of β-carotene concentration in hemp seeds and their breakdown in the gastrointestinal tract, absorption, storage in the liver, and transport to tissues. In cattle, an increased intake of β-carotene can lead to an increase in plasma retinol concentration, suggesting that vitamin A metabolism can be modulated through dietary interventions [41]. According to the literature, the retinol content of ruminant muscles may be significantly influenced by the composition of the feed ration. In a study of fallow deer (*Dama dama* L.), different diets induced significant variations in retinol levels in the longissimus muscle, even though β-carotene was not detected in the analyzed muscle. The cited authors demonstrated that dietary modifications may affect the conversion, transport, and retention of vitamin A in tissues, rather than merely the direct deposition of carotenoids [42].

An even more pronounced effect was observed for α-tocopherol. Its concentration increased from 0.029 mg/100 g of muscle tissue in CG to 0.139 and 0.169 mg/100 g of muscle tissue in groups HS–10 and HS–17, respectively (*p* < 0.001), corresponding to an approximately 4.8-fold and 5.8-fold increase, respectively. The observed differences could be due to the high α-tocopherol content of hemp seeds (6.17 mg/kg FM), substantially higher than that of ground triticale grain and rapeseed meal. Hemp seeds contain vitamin E and other antioxidants, but their profile varies depending on plant material and the production process [1,3,43]. Unlike retinol concentration, α-tocopherol concentration was significantly affected by experimental feeding and muscle type, as well as by the EF × MT interaction (*p* = 0.000, *p* = 0.000, and *p* = 0.007, respectively). Regardless of the feeding group, the SM muscle was characterized by a higher α-tocopherol concentration than the LL muscle (0.144 vs. 0.082 mg/100 g, respectively; Figure 2). In CG, the difference between the studied muscles was small. However, after the administration of hemp seeds, the response of the two tissues differed considerably. As a result, in the groups receiving hemp seeds, α-tocopherol concentration was approximately 1.6–2.1 times higher in the SM muscle than in the LL muscle. The retention of α-tocopherol in muscle tissues was not uniform and varied depending on the specific metabolic and structural properties of each muscle. In cattle supplemented with vitamin E, significant differences in α-tocopherol were found between muscles. The LL muscle accumulated less α-tocopherol than other muscles with different metabolic characteristics, despite identical supplementation levels [44]. A similar relationship was described in a previous study, where α-tocopherol concentration was higher in the SM muscle than in the LL muscle [45].

### 3.3. Effects of Experimental Feeding and Muscle Type on Intramuscular Fat Content and Fatty Acid Composition in Bulls

In the present study, the LL muscle had a significantly higher IMF content than the SM muscle (3.07% vs. 1.61%; *p* = 0.001), irrespective of the applied dietary treatment. These differences can be attributed to the distinct functions and metabolic characteristics of both muscles. Numerous studies have shown that bovine muscles differ in terms of the activity of energy pathways, structural organization, the proportion of muscle fibers, and chemical and lipid composition, which is reflected in their different IMF content and fatty acid profile [46,47]. Despite its substantially lower IMF content, the SM muscle contained more PUFAs (9.42% vs. 6.42%), including n-3 PUFAs (2.56% vs. 1.60%) and n-6 PUFAs (7.42% vs. 4.63%), than the LL muscle. A similar relationship was reported by Nogalski et al. [45], who compared these two muscles in young cattle: the SM muscle contained approximately 40% less IMF than the LL muscle, and it had a more favorable fatty acid profile. An increase in carcass fat content is accompanied by an increase in the amount of triacylglycerols stored in intramuscular adipocytes, which are relatively rich in SFAs and MUFAs. Changes in the content of membrane phospholipids are less significant; therefore, they account for a greater proportion of the total lipid pool in the leaner muscle. As phospholipids contain more PUFAs than triacylglycerols, the proportion of PUFAs expressed as a percentage of total fatty acids may be higher in a muscle with a lower IMF content. Similar observations were made in other ruminants [48].

The inclusion of whole hemp seeds in bull diets had no significant (*p* = 0.639) effect on the IMF content of the analyzed muscles, despite the observed decrease from 2.56% in CG to 2.32% and 2.11% in groups HS–10 and HS–17, respectively (Table 6). Similar results were reported by Klir et al. [5] and Muedi et al. [21] in their review articles on the use of industrial hemp in animal nutrition. The cited authors emphasized that hemp primarily modified the fatty acid composition, while its effect on the total fat content of tissues was minor and non-significant in most cases. Hemp seed supplementation had no influence on fat deposition in muscles, but it affected the quality of deposited lipids. The observed changes in the fatty acid profile suggest that the response to the applied dietary treatments involved the modification of muscle lipid composition, rather than an increase or a decrease in the total lipid pool.

An analysis of changes in the proportions of fatty acid groups in IMF revealed that a significant decrease in the proportion of SFAs, from 45.70% in CG to 40.33% in group HS–10 and 42.35% in group HS–17 (*p* = 0.001), was accompanied by an increase in the proportion of UFAs, from 54.33% to 59.67% and 57.65%, respectively (*p* = 0.001). Dietary supplementation with 10% hemp seeds produced the most favorable values for these fatty acid groups, whereas increasing the inclusion level to 17% did not result in a further improvement. Importantly, maize silage and total DMI were similar in HS–10 and HS–17 and concentrate DMI was virtually identical; therefore, this pattern is unlikely to be explained by a marked reduction in feed intake at the higher hemp seed inclusion level. The results of other experiments involving hemp-based products were similar. Increasing the proportion of the hemp component did not necessarily lead to a proportional improvement in the lipid profile of animal-based products [5,21]. In the current study, the proportion of n-6 PUFAs increased significantly (from 5.44% to 6.74%; *p* = 0.032), whereas the proportion of n-3 PUFAS remained unchanged (*p* = 0.337). As a result, the n-6/n-3 PUFA ratio was stable, ranging from 2.83 to 3.1. These findings suggest that feeding hemp seeds to bulls does not enrich beef with n-3 PUFAs, despite the fact that these seeds are a rich source of α-linolenic acid [3]. The lipid composition of feed does not directly translate into the lipid composition of ruminant meat, because a significant portion of UFAs undergo biohydrogenation in the rumen prior to absorption.

An analysis of the concentrations of individual fatty acids (Table 7) demonstrated that the observed reduction in the proportion of SFAs resulted primarily from decreasing levels of C16:0 and C18:0, followed by C10:0, C12:0, C15:0, C17:0, and C22:0. C16:0 is considered to exert a greater hypercholesterolemic effect than C18:0 [49]; therefore, its lower concentration can be beneficial from a nutritional perspective. The decrease in the content of this fatty acid was more pronounced at 10% than 17% inclusion of hemp seeds in bull diets, thereby confirming a non-linear response. In the group of MUFAs, the concentrations of C14:1, C17:1, and C18:1 c13 increased significantly, whereas the level of oleic acid (C18:1 c9), which is the most abundant MUFA in beef, remained unchanged. The above implies that the increase in the proportion of total MUFAs did not result from a marked increase in the concentration of a single dominant fatty acid. Instead, it was the outcome of minor alterations in multiple components. Significant changes were also observed in the trans isomers of oleic acid (C18:1). The proportion of total t6–t9 C18:1 increased in group HS–10, whereas the concentration of trans-vaccenic acid (TVA, C18:1 t11) decreased significantly from 1.62% in CG to approximately 1.01% in both experimental groups receiving hemp seeds (*p* = 0.001). No significant changes were found in the concentration of conjugated linoleic acid (CLA, c9,t11). In ruminant tissues, TVA is the primary substrate for the endogenous synthesis of cis-9, trans-11 CLA, catalyzed by Δ9-desaturase. The decrease in the concentration of TVA, with CLA content remaining unchanged, indicates that the increased supply of PUFAs with hemp seeds did not lead to the enhanced synthesis of this CLA isomer. The observed pattern may reflect changes in the formation and further conversion of specific biohydrogenation intermediates rather than differences in the overall extent of UFA metabolism. The relative production of trans-C18:1 intermediates, including TVA, is influenced by the lipid source and its physical form, diet composition, and the activity of ruminal microbial populations [3,5,21]. The mechanism responsible for the reduction in TVA content was not elucidated in this study. However, the administration of whole hemp seeds did not promote the enhancement of the pathway leading to TVA accumulation and subsequent CLA synthesis. The concentration of linoleic acid (LA, C18:2 n-6) increased from 4.45% in CG to 5.35% in group HS–17 (*p* = 0.044), which corresponded to the high LA content of hemp seeds (57.87% of total fatty acids). This observation confirms the partial transfer of dietary LA to muscle lipids despite biohydrogenation. The concentration of α-linolenic acid (ALA, C18:3 n-3) in muscle lipids decreased from 0.581% in CG to 0.427% and 0.424% in groups HS–10 and HS–17, respectively, although ALA accounted for 19.33% of total fatty acids in hemp seeds. This is a significant finding of the present study, which indicates that the high ALA content of the analyzed feed component does not translate directly into its high accumulation in ruminant meat. The limited transfer of dietary ALA into muscle lipids most likely reflects its low escape from ruminal metabolism before intestinal absorption. Consequently, a high ALA concentration in the diet does not necessarily result in a proportional increase in ALA available for deposition in muscle tissue [3,5,21]. It cannot be excluded that the partial replacement of rapeseed meal and ground triticale grain with hemp seeds affected the properties of the entire feed ration and the course of lipid metabolism in the rumen. The levels of eicosapentaenoic acid (EPA), docosapentaenoic acid (DPA), and docosahexaenoic acid (DHA) did not increase. The concentrations of EPA and DHA did not differ across the groups, and DPA concentration exhibited a non-significant numerical increase. These observations indicate that the increased dietary supply of ALA did not increase the proportion of long-chain n-3 PUFAs in beef. Effective enrichment of beef with n-3 PUFAs often requires protecting the fat source from ruminal breakdown or implementing strategies that influence the rumen microbiome [50].

The concentrations of individual fatty acids varied between the analyzed muscles. The SM muscle had higher concentrations of LA, c9,t11 CLA, arachidonic acid (AA), and long-chain n-3 PUFAs (EPA, DPA, and DHA), whereas the LL muscle had higher concentrations of myristic acid (C14:0), palmitic acid (C16:0), myristoleic acid (C14:1), and total t6-t9 C18:1. These results are consistent with the previously observed differences in IMF content and the proportion of total PUFAs, and suggest that muscle lipid composition is largely determined by the metabolic properties of each muscle. Muscles that differ in fat content and the proportion of structural lipids are characterized by different fatty acid profiles, particularly with regard to total PUFAs and long-chain n-3 PUFAs [10,13]. A significant EF × MT interaction was found for the concentrations of C10:0, C17:0, total t6-t9 C18:1, AA, DPA, and DHA, which indicates that the responses of the SM muscle and the LL muscle to dietary hemp seed supplementation were not identical for all analyzed fatty acids. A similar phenomenon has been previously described in cattle, where the effect of diet on the fatty acid profile varied across the studied muscles [45,51]. These findings suggest that the efficacy of dietary modification of muscle lipid composition may be related to differences in the organization of lipid fractions and the metabolic properties of individual muscles. However, further research is needed to elucidate the mechanisms responsible for the observed differences.

A limitation of the present study was the lack of independent pen-level replication, as each dietary treatment was represented by a single pen. Therefore, potential pen effects could not be separated from dietary treatment effects. Nevertheless, feed intake and meat composition traits were recorded at the individual-animal level, providing eight biological observations per treatment. This sample size is consistent with that used in controlled cattle feeding studies; however, statistical power to detect small effects may have been limited for fatty acids occurring at low proportions. Because inclusion levels below 10% were not evaluated, the present experiment does not allow determination of an optimal or minimum effective dietary inclusion level. Therefore, the observed changes in selected fatty acid, vitamin, and mineral traits should be interpreted as responses obtained under the specific experimental conditions. Further studies including more evenly distributed hemp seed inclusion levels, particularly levels below 10%, would help to more precisely determine the minimum effective inclusion level in cattle diets.

## 4. Conclusions

The results of this study indicate that whole hemp seeds can be a valuable component of beef cattle diets, modifying selected nutritional characteristics of beef. Hemp seed inclusion increased the concentrations of Zn, Mg, Fe, retinol, and α-tocopherol in muscle tissue and modified the fatty acid profile by decreasing the proportion of SFAs and increasing the proportion of UFAs and the PUFA/SFA ratio. However, the concentrations of EPA, DPA, and DHA did not increase despite the high ALA content of hemp seeds. For several measured traits, the responses observed at 10% inclusion were not further enhanced when the inclusion level was increased to 17%. However, because inclusion levels below 10% were not evaluated, the present results do not establish 10% as the optimal or minimum effective level. Overall, whole hemp seeds show potential as a feed ingredient for improving selected nutritional characteristics of beef. Further dose–response studies, including inclusion levels below 10%, are needed to determine the most effective dietary level.

## Figures and Tables

**Figure 1:**
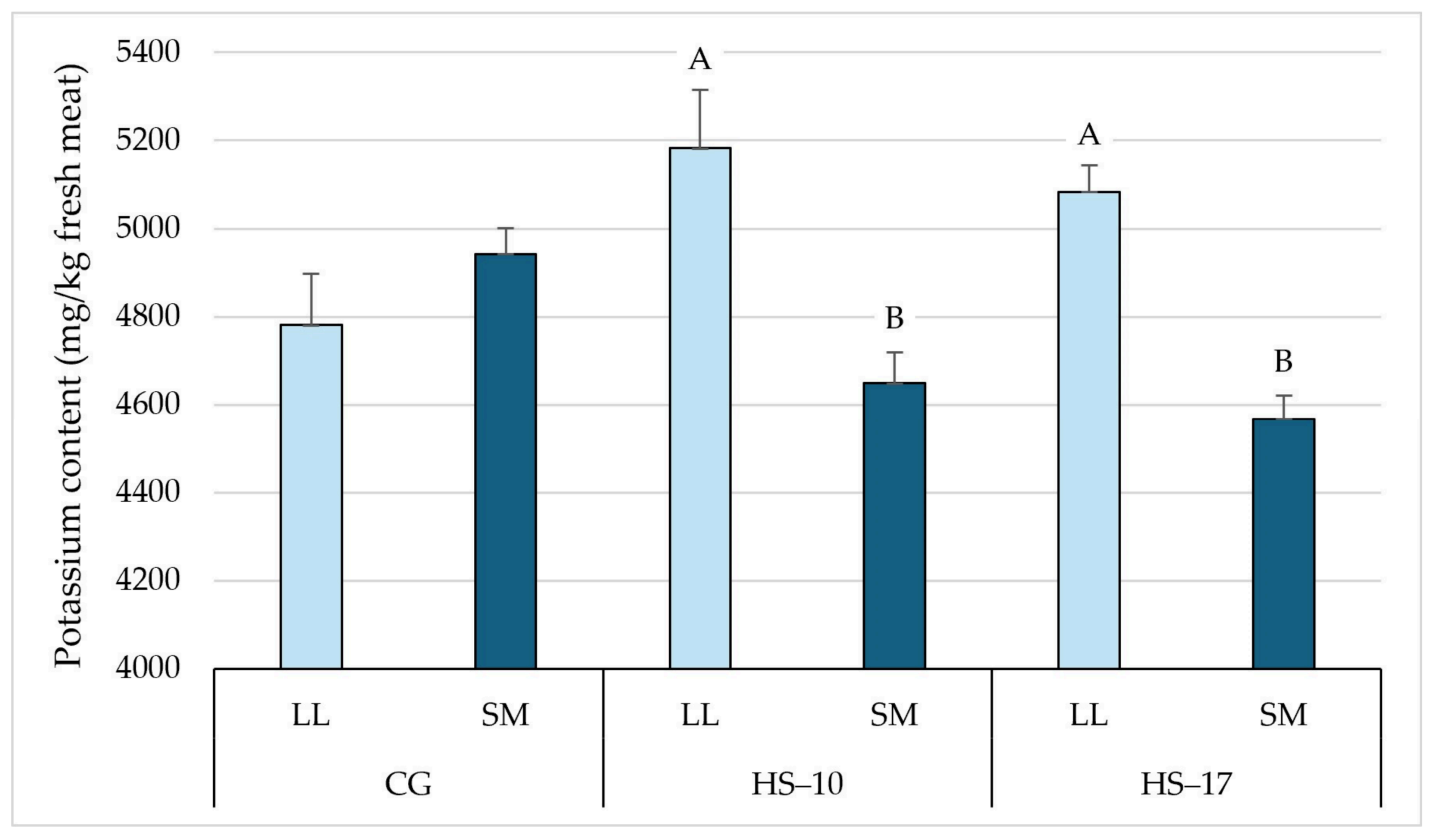
The interaction effect of experimental feeding x muscle type on the potassium content of beef (vertical bars represent the standard error of the mean, SEM; A, B (*p* ≤ 0.01); CG—control group receiving a diet without hemp (*Cannabis sativa* L.) seeds; HS–10—experimental group receiving a diet supplemented with 10% hemp seeds; HS–17—experimental group receiving a diet supplemented with 17% hemp seeds; LL, longissimus lumborum muscle; SM, semimembranosus muscle.

**Figure 2:**
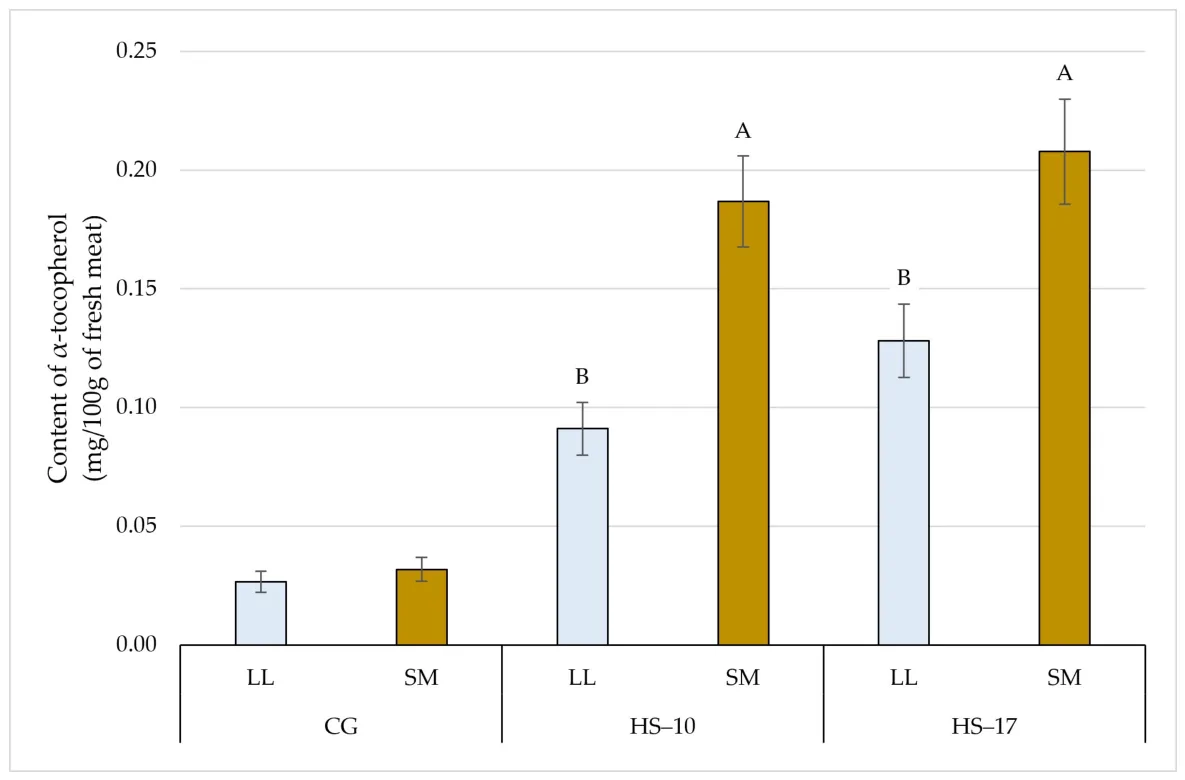
The interaction effect of experimental feeding x muscle type on the α-tocopherol content of beef (vertical bars refer to the standard error of the mean, SEM; A, B (*p* ≤ 0.01); CG—control group receiving a diet without hemp (*Cannabis sativa* L.) seeds; HS–10—experimental group receiving a diet supplemented with 10% hemp seeds; HS–17—experimental group receiving a diet supplemented with 17% hemp seeds; LL, *longissimus lumborum* muscle; SM, *semimembranosus* muscle.

**Table 1:** Chemical composition and nutritional value of experimental diets (mean ± standard error).

Specification	Maize Silage	Ground Triticale Grain	Rapeseed Meal	*Cannabis sativa* L. Seeds
DM, g∙kg^−1^ FM	375 ± 1	877 ± 1	880 ± 1	877 ± 1
Chemical composition, g·kg^−1^ DM				
Organic matter	969 ± 2	966 ± 1	927 ± 2	940 ± 2
Crude protein	78 ± 1.2	136 ± 1	395 ± 2	244 ± 2
Crude fat	21 ± 06	12 ± 1	18 ± 1	301 ± 2
NDF	336 ± 4	172 ± 1	298 ± 2	310 ± 2
ADF	194 ± 1	42 ± 0	212 ± 2	231 ± 1
NFC	612 ± 4	646 ± 4	215 ± 3	85 ± 0.4
pH	3.7 ± 0.08			
Lactic acid	27.8 ± 7.1			
Acetic acid	6.3 ± 1.2			
Butyric acid	0.08 ± 0.03			
N-NH3 (g kg^−1^ TN)	33.6 ± 11.4			
UFV	0.85 ± 0.01	1.19 ± 0.01	1.05 ± 0.01	1.62 ± 0.01
PDIN	48 ± 0.5	94 ± 0.1	257 ± 1	161 ± 04
PDIE	69 ± 0.6	109 ± 0.2	159 ± 1	95 ± 0.5
Fatty acid profile (g/100 g fatty acids)				
C 14:0	0.15 ± 0.01	0.19 ± 0.01	0.29 ± 0.01	0.04 ± 0.00
C 16:0	13.79 ± 0.67	18.49 ± 0.03	10.86 ± 0.03	6.60 ± 0.04
C 18:0	1.95 ± 0.09	1.34 ± 0.02	2.68 ± 0.01	3.24 ± 0.03
C18:1 n-9 (OA)	23.63 ± 0.42	14.00 ± 0.06	43.82 ± 0.11	10.99 ± 0.08
C18:2 n-6 (LA)	51.56 ± 0.12	58.96 ± 0.14	31.04 ± 0.06	57.87 ± 0.04
C18:3 n-3 (ALA)	7.77 ± 0.08	6.23 ± 0.03	7.39 ± 0.014	19.33 ± 0.15
Vitamin content, mg∙kg^−1^ FM				
β- carotene	18.20 ± 2.89	0.87 ± 0.06	0.81 ± 0.04	5.69 ± 1.19
α-tocopherol	0.20 ± 0.04	0.68 ± 0.01	0.29 ± 0.01	6.17 ± 1.57

DM—dry matter; FM—fresh matter; NDF—neutral detergent fiber; ADF—acid detergent fiber; NFC—non-fiber carbohydrate; TN—total nitrogen; UFV—feed units for meat production; PDIN—protein digested in the small intestine when rumen-degradable nitrogen is limiting; PDIE—protein digested in the small intestine when rumen-fermentable energy is limiting.

**Table 2:** Ingredients (% dry matter, DM) and chemical composition of the concentrate and mean daily dry matter intake of bulls in the experimental groups. (mean ± standard error).

Specification	CG	HS–10	H–17
Maize silage	*ad libitum*	*ad libitum*	*ad libitum*
Concentrated feed:			
- ground triticale grain	79	73	69
- rapeseed meal	18	14	11
- hemp (*Cannabis sativa* L.) seeds	0	10	17
Premix ^1^	3	3	3
Chemical composition and nutritional value of concentrated feed (g·kg^−1^ DM)			
Dry matter, g·kg^−1^ FM	851.2	851.1	851.0
Organic matter	930.0	929.0	928.3
Crude protein	178.5	179.0	178.8
Crude fat	12.7	41.3	61.4
NDF	189.5	198.3	204.2
ADF	71.3	83.4	91.6
NFC	549.0	510.2	483.8
UFV	1.1	1.2	1.2
PDIN	120.5	120.7	120.5
PDIE	114.7	111.3	108.9
Dry matter intake, kg DM/day			
Maize silage	7.224	8.771	8.629
Concentrated feed	4.256	4.256	4.255
Total	11.480	13.027	12.884

CG—control group receiving a diet without hemp seeds, HS–10—experimental group receiving a diet supplemented with 10% hemp seeds, HS–17—experimental group receiving a diet supplemented with 17% hemp seeds, FM—fresh matter, NDF—neutral detergent fiber; ADF—acid detergent fiber; NFC—non-fiber carbohydrate; UFV—feed units for meat production, PDIN—protein digested in the small intestine when rumen-degradable nitrogen is limiting; PDIE—protein digested in the small intestine when rumen-fermentable energy is limiting; ^1^ Commercial mineral–vitamin premix for beef cattle (915926-W25 Bestermine fattening; De Heus Animal Nutrition, Łęczyca, Poland) consisting of: Ca, 19%; Na, 7%; P, 6%; Mg, 5%; K, 01%; vitamin A, 500,000.00 IU/kg; vitamin D3, 1,000,000.00 IU/kg; vitamin E, 3500.00 mg/kg; Cu, 1000.00; I, 80.00 mg/kg; Mn, 1600.00 mg/kg; Fe, 400.00 mg/kg; Se, 10.00 mg/kg; Co, 80.00 mg/kg; Zn, 3000.00 mg/kg.

**Table 3:** The operating conditions of the atomic absorption spectrometer used for mineral analysis.

Elements	Fuel	Support	Wavelength	Spectral Bandpass
Potassium (K)	Acetylene	Air	766.5 nm	0.2 nm
Sodium (Na)	Acetylene	Air	589.0 nm	0.2 nm
Magnesium (Mg)	Acetylene	Air	202.6 nm	1.0 nm
Zinc (Zn)	Acetylene	Air	213.9 nm	1.0 nm
Iron (Fe)	Acetylene	Air	248.3 nm	0.2 nm

**Table 4:** Mineral (mg/kg fresh meat) content of the *longissimus lumborum* and *semimembranosus* muscles of bulls.

Specification	Experimental Feeding (EF)			Muscle Type (MT)		SEM	*p*-Value		
	CG (*n* = 8)	HS–10 (*n* = 8)	HS–17 (*n* = 8)	LL (*n* = 24)	SM (*n* = 24)		EF	MT	EF × MT
Zn	40.87 ^Bb^	50.75 ^a^	53.04 ^A^	54.10 ^A^	42.54 ^B^	14.356	0.013	0.001	0.060
Mg	208.63 ^B^	240.24 ^A^	246.55 ^A^	233.66	230.63	23.647	0.000	0.581	0.271
Fe	21.86 ^b^	25.43 ^a^	26.00 ^a^	25.80 ^a^	23.14 ^b^	5.080	0.025	0.054	0.062
K	4862.08	4916.20	4825.54	5022.56 ^A^	4717.17 ^B^	332.778	0.588	0.000	0.000
Na	582.06	578.25	587.19	603.35 ^a^	561.30 ^b^	68.563	0.928	0.039	0.462

CG—control group receiving a diet without hemp (*Cannabis sativa* L.) seeds; HS–10—experimental group receiving a diet supplemented with 10% hemp seeds; HS–17—experimental group receiving a diet supplemented with 17% hemp seeds; LL, *longissimus lumborum* muscle; SM, *semimembranosus* muscle; SEM, standard error of the mean; Zn–zinc; Mg–magnesium; Fe–iron; K–potassium; Na–sodium; means followed by different superscript letters differ within rows and within the same factor. Uppercase letters (A, B) indicate differences significant at *p* ≤ 0.01, whereas lowercase letters (a, b) indicate differences significant at *p* ≤ 0.05.

**Table 5:** Effects of experimental feeding and muscle type on the content of retinol and α-tocopherol in the *longissimus lumborum* and *semimembranosus* muscles of bulls (mg/100 g fresh meat).

Specification	Experimental Feeding (EF)			Muscle Type (MT)		SEM	*p*-Value		
	CG (*n* = 8)	HS–10 (*n* = 8)	HS–17 (*n* = 8)	LL (*n* = 24)	SM (*n* = 24)		EF	MT	EF × MT
Retinol	0.0059 ^Aa^	0.013 ^B^	0.018 ^b^	0.014	0.011	0.009	0.001	0.190	0.555
α-tocopherol	0.029 ^B^	0.139 ^A^	0.169 ^A^	0.082 ^B^	0.144 ^A^	0.080	0.000	0.000	0.007

CG—control group receiving a diet without hemp (*Cannabis sativa* L.) seeds; HS–10—experimental group receiving a diet supplemented with 10% hemp seeds; HS–17—experimental group receiving a diet supplemented with 17% hemp seeds; LL, *longissimus lumborum* muscle; SM, *semimembranosus* muscle; SEM, standard error of the mean; means followed by different superscript letters differ within rows and within the same factor. Uppercase letters (A, B) indicate differences significant at *p* ≤ 0.01, whereas lowercase letters (a, b) indicate differences significant at *p* ≤ 0.05. A mean may carry both uppercase and lowercase letters when separate pairwise comparisons involving that mean reach different significance levels.

**Table 6:** Effects of experimental feeding and muscle type on intramuscular fat content and the percentages and ratios of fatty acid groups in the intramuscular fat of bulls (%, g/100 g total fatty acids).

Specification	Experimental Feeding (EF)			Muscle Type (MT)		SEM	*p*-Value		
	CG (*n* = 8)	HS–10 (*n* = 8)	HS–17 (*n* = 8)	LL (*n* = 24)	SM (*n* = 24)		EF	MT	EF × MT
IMF, %	2.559	2.315	2.109	3.073 ^A^	1.612 ^B^	0.217	0.639	0.000	0.239
SFAs	45.704 ^A^	40.332 ^B^	42.349 ^B^	43.540	41.981	0.533	0.000	0.087	0.168
UFAs	54.330 ^B^	59.667 ^A^	57.649 ^A^	56.480	58.019	0.533	0.000	0.093	0.160
MUFAs	46.857 ^Bb^	51.885 ^A^	49.048 ^a^	50.064	48.599	0.520	0.000	0.076	0.551
PUFAs	7.473	7.782	8.601	6.416	9.420	0.310	0.079	0.000	0.075
MUFA/SFA	1.034 ^A^	1.303 ^B^	1.163	1.152	1.159	0.028	0.000	0.970	0.350
PUFA/SFA	0.166 ^b^	0.194	0.206 ^a^	0.148	0.227	0.008	0.016	0.000	0.026
n-3	1.978	2.049	2.249	1.602	2.561	0.107	0.337	0.000	0.060
n-6	5.439 ^b^	5.985	6.738 ^a^	4.631	7.417	0.292	0.032	0.000	0.059
n-6/n-3	2.825	3.030	3.114	3.062	2.922	0.075	0.279	0.353	0.343

CG—control group receiving a diet without hemp (*Cannabis sativa* L.) seeds, HS–10—experimental group receiving a diet supplemented with 10% hemp seeds, HS–17—experimental group receiving a diet supplemented with 17% hemp seeds; LL, *longissimus lumborum* muscle; SM, *semimembranosus* muscle; SFAs—saturated fatty acids; UFAs—unsaturated fatty acids; MUFAs—monounsaturated fatty acids; PUFAs—polyunsaturated fatty acids; SEM, standard error of the mean; means followed by different superscript letters differ within rows and within the same factor. Uppercase letters (A, B) indicate differences significant at *p* ≤ 0.01, whereas lowercase letters (a, b) indicate differences significant at *p* ≤ 0.05. A mean may carry both uppercase and lowercase letters when separate pairwise comparisons involving that mean reach different significance levels.

**Table 7:** Effects of experimental feeding and muscle type on the percentages of fatty acids in intramuscular fat (%, g/100 g total fatty acids).

Specification	Experimental Feeding (EF)			Muscle Type (MT)		SEM	*p*-Value		
	CG (*n* = 8)	HS–10 (*n* = 8)	HS–17 (*n* = 8)	LL (*n* = 24)	SM (*n* = 24)		EF	MT	EF × MT
C 10:0	0.037 ^A^	0.028 ^B^	0.025 ^B^	0.049 ^A^	0.026 ^B^	0.001	0.000	0.000	0.009
C 12:0	0.055 ^A^	0.040 ^B^	0.041 ^B^	0.049 ^a^	0.041 ^b^	0.002	0.000	0.003	0.157
C 14:0	2.542 ^a^	2.136 ^b^	2.289	2.548 ^A^	2.098 ^B^	0.072	0.047	0.001	0.952
C 14:1	0.475 ^Bb^	0.747 ^A^	0.703 ^a^	0.770 ^A^	0.523 ^B^	0.041	0.003	0.001	0.486
C 15:0	0.394 ^A^	0.300 ^B^	0.301 ^B^	0.330	0.332	0.010	0.000	0.795	0.389
C 16:0	25.710 ^Aa^	23.559 ^B^	24.111 ^b^	25.208 ^A^	23.706 ^B^	0.307	0.006	0.008	0.322
C 16:1	3.351	4.209	3.808	4.281	3.334	0.202	0.165	0.016	0.580
C 17:0	1.033 ^a^	0.930	0.866 ^b^	0.957	0.929	0.028	0.032	0.640	0.020
C17:1	0.743 ^b^	0.831 ^a^	0.786	0.782	0.793	0.015	0.055	0.762	0.271
C 18:0	15.395 ^A^	12.953 ^B^	14.346	13.969	14.432	0.322	0.006	0.393	0.469
C 18:1 T6+9	0.578 ^B^	1.476 ^A^	1.056	1.484 ^A^	0.624 ^B^	0.146	0.005	0.000	0.003
C 18:1 (TVA)	1.623 ^A^	1.005 ^B^	1.032 ^B^	0.938 ^B^	1.482 ^A^	0.086	0.000	0.000	0.480
C 18:1 C9 (OA)	37.445	39.051	38.682	37.981	38.814	0.478	0.380	0.421	0.094
C 18:1 C11	1.665	3.415	1.882	2.764	1.939	0.363	0.065	0.230	0.130
C 18:1 C12	0.265	0.307	0.277	0.289	0.278	0.015	0.419	0.712	0.058
C 18:1 C13	0.288 ^b^	0.387 ^a^	0.356	0.338	0.351	0.015	0.026	0.724	0.931
C 18:1 T16	0.318 ^a^	0.256 ^b^	0.278	0.272	0.295	0.008	0.004	0.093	0.223
C 18:2 (LA)	4.445 ^b^	4.752	5.345 ^a^	3.799 ^B^	5.850 ^A^	0.214	0.044	0.000	0.097
C 18:2 c9 t11	0.602	0.572	0.588	0.562 ^B^	0.612 ^A^	0.022	0.316	0.000	0.288
C 18:3 (LNA)	0.581 ^a^	0.427 ^b^	0.424 ^b^	0.472	0.480	0.026	0.020	0.827	0.441
C 20:4 (AA)	0.994 ^b^	1.233	1.394 ^a^	0.832	1.567	0.082	0.025	0.000	0.030
C 20:5	0.106	0.085	0.102	0.074	0.120	0.006	0.139	0.000	0.135
C 22:0	0.062 ^a^	0.040 ^b^	0.040 ^b^	0.035	0.058	0.004	0.005	0.000	0.750
C 22:5 (DPA)	0.259	0.270	0.293	0.200	0.345	0.016	0.436	0.000	0.032
C 22:6 (DHA)	0.039	0.034	0.036	0.024	0.049	0.003	0.481	0.000	0.012

CG—control group receiving a diet without hemp (*Cannabis sativa* L.) seeds, HS–10—experimental group receiving a diet supplemented with 10% hemp seeds, HS–17—experimental group receiving a diet supplemented with 17% hemp seeds; LL, *longissimus lumborum* muscle; SM, *semimembranosus* muscle; TVA—trans-vaccenic acid; OA—oleic acid; LA—linoleic acid; CLA—conjugated linoleic acid; LNA—linolenic acid; AA—arachidonic acid; EPA—eicosapentaenoic acid; DPA—docosapentaenoic acid; DHA—docosahexaenoic acid; SEM, standard error of the mean; means followed by different superscript letters differ within rows and within the same factor. Uppercase letters (A, B) indicate differences significant at *p* ≤ 0.01, whereas lowercase letters (a, b) indicate differences significant at *p* ≤ 0.05. A mean may carry both uppercase and lowercase letters when separate pairwise comparisons involving that mean reach different significance levels.

## Data Availability

The raw data supporting the conclusions of this article will be made available by the authors on request.

## Abbreviations

The following abbreviations are used in this manuscript:

AA	arachidonic acid
ADF	acid detergent fiber
ALA	α-linolenic acid
ANOVA	analysis of variance
CG	control group
CLA	conjugated linoleic acid
DHA	docosahexaenoic acid
DM	dry matter
DPA	docosapentaenoic acid
EF	experimental feeding
EPA	eicosapentaenoic acid
FAME	fatty acid methyl ester
FM	fresh matter
GC	gas chromatography
GC-FID	gas chromatography–flame ionization detection
HPLC	high-performance liquid chromatography
HS–10	experimental group receiving a diet supplemented with 10% hemp seeds
HS–17	experimental group receiving a diet supplemented with 17% hemp seeds
HSD	honestly significant difference
IMF	intramuscular fat
LA	linoleic acid
LL	*longissimus lumborum muscle*
LW	live weight
MT	muscle type
MUFA	monounsaturated fatty acid
NDF	neutral detergent fiber
NFC	non-fiber carbohydrate
OA	oleic acid
PDIE	protein digested in the small intestine when rumen-fermentable energy is limiting
PDIN	protein digested in the small intestine when rumen-degradable nitrogen is limiting
HF	Holstein-Friesian
PUFA	polyunsaturated fatty acid
RIC	Roughage Intake Control
RP-HPLC	reversed-phase high-performance liquid chromatography
SEM	standard error of the mean
SFA	saturated fatty acid
SM	*semimembranosus muscle*
TMR	total mixed ration
TN	total nitrogen
TVA	trans-vaccenic acid
UFA	unsaturated fatty acid
UFV	feed units for meat production
